# Therapeutic potential of adipose-derived stem cells for diabetic foot ulcers: a systematic review and meta-analysis

**DOI:** 10.1186/s13098-024-01523-5

**Published:** 2025-01-07

**Authors:** Mohamed A. Abu Elainein, Mohamad Mahmoud Whdan, Mahmoud Samir, Nada G. Hamam, Mohamed Mansour, Mohamed Abdel Mohsen Mohamed, Mahmoud Mostafa Snosy, Mahmoud Ayman Othman, Ahmed Sayed Sobieh, Mahmoud Gamal Saad, Mohamed A. Labna, Salma Allam

**Affiliations:** 1 Faculty of Medicine, Ain Shams University, Cairo, Egypt; 2 Faculty of Medicine, Cairo University, Cairo, Egypt; 3 Faculty of Medicine, Galala University, Suez, Egypt

**Keywords:** Diabetes, Diabetic foot ulcers, Stem cells, Adipose stem cells, Regenerative medicine

## Abstract

**Background:**

As the global prevalence of diabetes mellitus increases, the incidence of non-healing wounds in diabetic patients is expected to rise significantly, according to the International Diabetes Federation (IDF), around 537 million adults currently suffer from diabetes mellitus worldwide and 20% to 30% of individuals with diabetes are hospitalized due to diabetic foot ulcers. Conventional treatments such as traditional dressings often fall short in ensuring satisfactory wound healing, this Meta-analysis investigates the therapeutic potential of Adipose-derived Stem Cells (ADSCs) as a promising strategy for addressing this challenge.

**Aims:**

To Assess the Therapeutic Potential of Adipose-Derived Stem Cells for Managing Diabetic Foot Ulcers compared to conventional lines of treatments.

**Methods:**

The PubMed, SCOPUS, Web of Science Core Collection, Cochrane Library, and ClinicalTrials.gov. databases were searched from January 2000 and December 2023, articles were primarily evaluated regarding their titles and abstracts, then full-text screening was assessed against the inclusion and exclusion criteria by utilizing Rayyan software. The Cochrane risk of bias (RoB 2) assessment tool was used to identify the risk of bias in our included studies. A statistical analysis was performed using Review Manager (RevMan) Version 5 software. Dichotomous data was subjected to risk ratio analysis, while continuous data underwent Mean Difference (MD) evaluation, all was reported with 95% confidence intervals, P value is considered statistically significant if less than 0.05.

**Results:**

Regarding the total healing state, five studies reported that more participants healed completely at the end of the follow-up period in the ADSCs group (Risk ratio = 1.56, 95% CI [1.32, 1.86], P < 0.00001), for the healing rate the overall effect estimate favors the ADSCs group (pooled effect estimate = 1.84, 95% CI [1.51, 2.89], P < 0.00001), and regarding the healing time the pooled mean difference of the studies demonstrated that the ADSCs group required fewer days to heal than the standard care group. (pooled mean difference = −19.33, 95% CI [−37.36, −1.29], P = 0.04).

**Conclusion:**

ADSCs provide favorable healing results and safety compared to standard care for diabetic foot ulcers.

**Supplementary Information:**

The online version contains supplementary material available at 10.1186/s13098-024-01523-5.

## Introduction

One of the most serious diabetic complications is Diabetic Foot Ulcers (DFUs), 20–30% of diabetic patients are hospitalized due to diabetic foot ulcers, and every year, out of the increased 5% of diabetes mellitus there is 1% reported for amputation due to diabetic foot ulcer [1, 2].

DFUs remain an issue for healthcare systems. The growing prevalence of diabetes along with changes in demographics and lifestyles underscores the need for solutions to prevent and treat DFUs. The long-term nature of DFUs requires management that places the burden on patients, caregivers, and healthcare providers [3]. It represents a real challenge due to their tendency for chronicity, and susceptibility to infections, it also increases the risk of lower limb amputations, additionally, it has economic and financial burdens primarily driven by the costs of medications and the high rate of hospitalizations between the patients [4, 5]. Neuropathic and ischemic complications of DM represent 90% of DFUs and the remaining 10% are attributed to ischemic ulcers associated with DM [6].

The standard treatment of DFU involves standard care procedures for prevention of progress and giving a chance for healing like debridement, dressing, and infection control in addition to glycemic control which is the main stone of treatment initiation and adjuvant therapies that accelerate the process of healing and give a better cosmetic effect that include some topical agents like iodine, negative pressure wound therapy, skin grafts, human growth factors. This strategy encompasses not only treatments but also patient education as a vital component, making lifestyle changes, and coordinating care across different healthcare facilities, however successfully managing DFUs demands collaboration from healthcare professionals and policymakers [7–9].

Recently, Mesenchymal stem cells (MSCs) have been used for the treatment of DFUs. Mesenchymal stem cells (MSCs) can differentiate into multiple cell types which is crucial for tissue regeneration, including neurogenic, chondrogenic, adipogenic, osteogenic, myogenic, and endothelial cells, taking MSCs from various tissue origins such as bone marrow and umbilical cord blood is relatively straightforward, and they can be efficiently expanded in culture [10].

Among the multiple types of MSCs, Adipose-Derived Stem Cells (ADSCs) have distinctive characteristics and great therapeutic potential [10]. ADSCs can differentiate into several cell lineages; they possess immune-modulatory properties, secrete bioactive factors facilitating repair, and enhance human growth factors and regeneration of tissues [10, 11].

ADSCs are extracted from a brown or white adipocyte that is abundant in our bodies, it can be obtained by a simple invasive surgery from subcutaneous tissues of many sites like the abdomen, thigh, and buttocks. The high concentration of stem cells in adipocytes (about 100 ml can be collected from 1000 ml adipose tissue) gives ADSCs an advantage over other types of stem cells [12, 13].

Despite the frequent use of ADSCs, there is limited data on the comparative effectiveness between ADSC-based therapies versus conventional wound care modalities in DFU treatments.

To address this objective, it's crucial to conduct a meta-analysis comparing ADSC-based interventions with traditional wound care methods to fill knowledge gaps and improve patient outcomes.

## Methods

### Protocol registration

We have registered our meta-analysis protocol to the International Prospective Register of Systematic Reviews (PROSPERO). The study's registration number is [CRD42023484473].

### Literature search

Adherence to the Recommended Reporting Items for Systematic Reviews and Meta-analyses (PRISMA) guidelines and utilizing the PICOS model for inclusion and exclusion criteria. A systematic review of the literature was conducted, our search strategy included five databases [PubMed, Web of Science Core Collection, SCOPUS, Cochrane Library, and ClinicalTrials.gov.] and the publication was limited to English language only, published between January 2000 and December 2023 [14].

Our inclusion criteria include Randomized Controlled trials that establish the role of ADSCs in the management of DFUs including all genders and age groups published in the English language, while exclusion criteria involve observational studies, non-randomized Controlled trials, in vitro studies, animal studies, and articles published in languages other than English.

We have searched these different databases by the following search terms:

("adipose-derived stem cells" OR "stem cell therapy" OR "adipose stem cells" OR "ADSCs" OR "adipose-derived stromal cells" OR "mesenchymal stem cells" OR "fat graft") AND ("diabetic foot ulcer" OR "DFU" OR "Diabetic injury" OR "diabetic leg ulcer" OR "diabetic wound" OR “diabetic foot” OR “diabetic leg”).

**Fig. 1:**
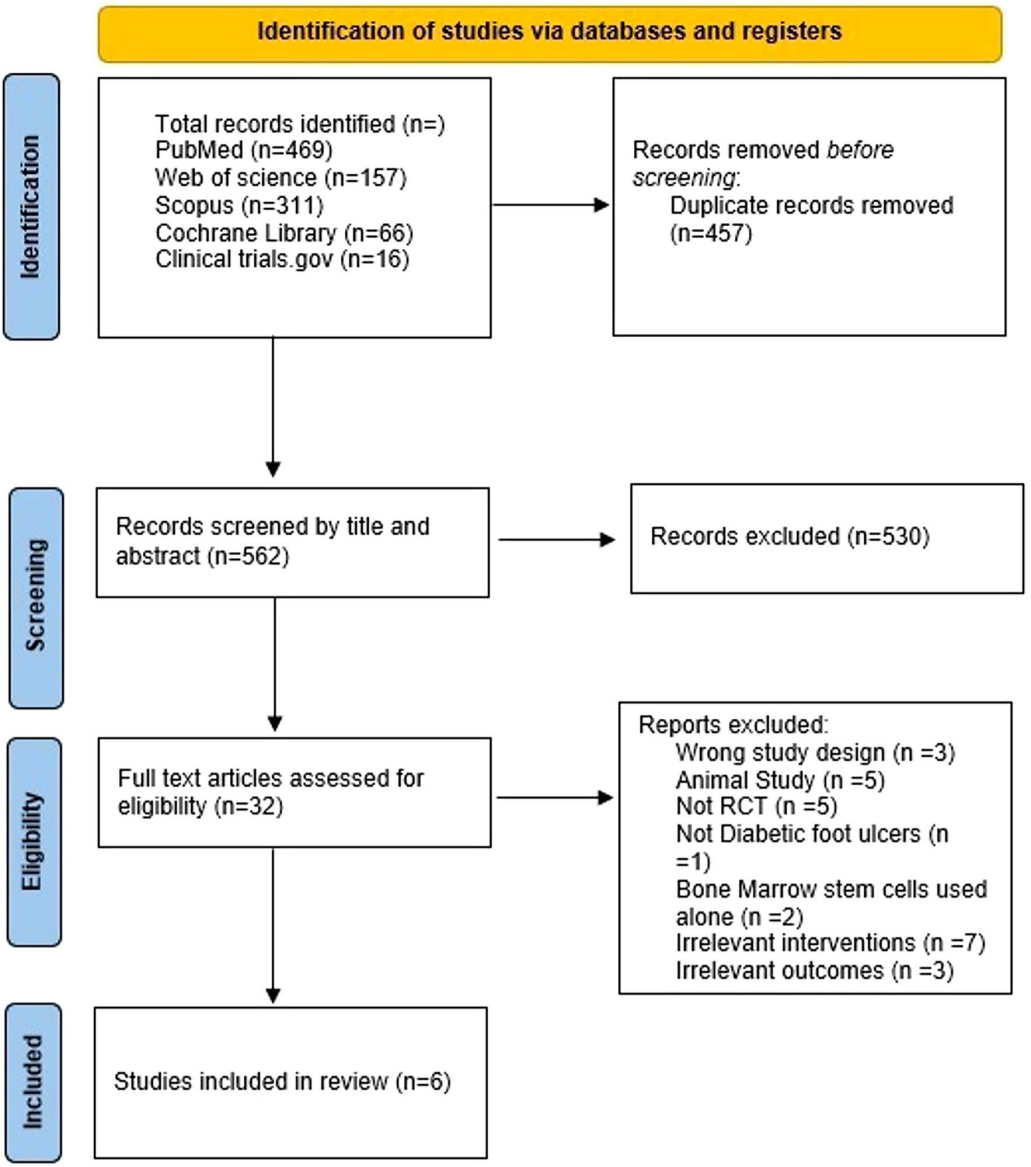
PRISMA flowchart

Study selection involved two independent reviewers, with a third reviewer resolving conflicts, articles were primarily evaluated regarding their titles and abstracts, and then full-text screening was assessed against the inclusion and exclusion criteria by utilizing Rayyan software, the screening process details are available in PRISMA flowchart (Fig. 1) [15].

### Data extraction strategy

Data extraction was performed and verified independently by study authors and an Excel sheet was used for extraction of data like demographic data of the population, baseline characteristics of ulcers, comorbidities in the study population, data about study location, sample size, inclusion and exclusion criteria, duration of the study, duration of follow-up and outcome indicators.

The primary outcomes we targeted included healing rate, healing time, and the total number of healed ulcers at the end of follow-up duration and the secondary outcomes were relative ulcer size, pain level, cost-effectiveness, histological assessment of the ulcer, and how the intervention improves the quality of life for the included study groups.

### Risk of bias assessment

**Fig. 2:**
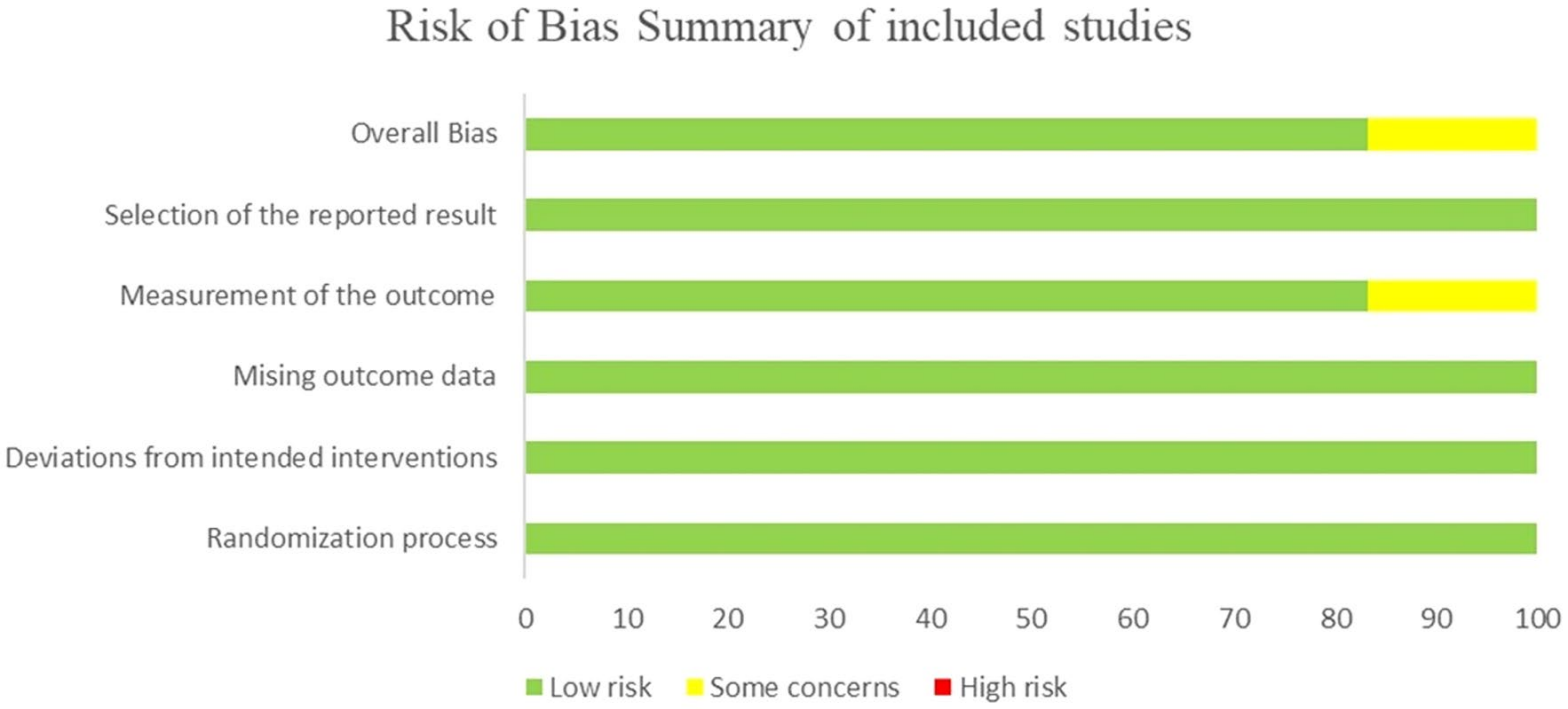
Risk of bias summary and graph of the six included studies based on the Cochrane risk of bias assessment tool (ROB 2 tool)

We used the Cochrane Risk of Bias tool () (RoB 2) to assess the bias in our included studies through its several domains. the risk of bias was assessed by two independent reviewers as they mutually cross-checked the quality of each study to give a decision and a third reviewer took the final decision if there was any conflict, graphs representing the risk of bias in our included studies are shown in Fig. 2, The Grading of Recommendations Assessment, Development, and Evaluation (GRADE) system was used to investigate the level of the evidence [16].

### Statistical analysis

A statistical analysis was conducted using Review Manager (RevMan) Version 5 software. Dichotomous data was subjected to Risk ratio analysis, while continuous data underwent Mean Difference (MD) evaluation, all were reported with 95% confidence intervals. Heterogeneity among the included studies was assessed through the I^2^ test. The result is considered statistically significant if less than 0.05. In instances where I^2^ exceeds 50%, a Random Effect Model was employed; otherwise, the Fixed Effects Model was used [16].

## Results

### Search results and study characteristics

The most recent version of the PRISMA flow chart demonstrates the methodology used in our study for screening relevant searches (Fig. 1). The total number of identified potentially related records was 1019 records from which there were 457 duplicate records removed. 530 records were excluded according to their irrelevant title and abstracts and 32 full texts were assessed for eligibility criteria resulting in the exclusion of 26 records and the inclusion of 6 records (Fig. 1).

The six included studies were published between 2019 and 2023. All the studies are randomized controlled trials, with f 316 participants (162 in the adipose stem cells group and 154 in the standard care group). These studies were conducted in different countries (Egypt, Italy, Turkey, Germany, the UK, and Korea) (23–28). The summary of the included studies and the follow-up durations are presented in Table 1. The participants’ mean age ranged from 48.04 to 71.6 years, and the proportion of males ranged from 48 to 92.9%. The participant demographics and baseline characteristics are shown in Table 2 [17–22].

**Table 1:** Summary of the included studies

No	Study ID	Country	Study design	Total sample size (n)	N of Pts for arm		Duration of the study (MONTH)	Duration of follow up (DAY)	Outcome indicators	Type of ADSC-based intervention	Characteristics of the treated ulcers
					ADSCs group	Standard care group					
1	Tanios (2021) [17]	Egypt	Randomized controlled trial	100	50	50	29	180	Ulcer size, histological features, overall healing results, clinical parameters of ulcer, stem cell yield viability, Ulcer preparation for further intervention	Autologous adipose-derived stromal vascular fraction cells	Chronic ulcers, including diabetic foot ulcers and trophic ulcers
2	Lonardi (2019) [18]	Italy	Randomized controlled trial	114	57	57	36	180	Healing rate, healing time, pain level, relapse rate, the skin tropism	Autologous micro-fragmented adipose tissue	Irreversible digital/forefoot ulcer/gangrene (with negative X-ray for osteolytic lesions) following minor amputation due to complications of DFU
3	Uzun (2021) [19]	Turkey	Randomized controlled trial	20	10	10		1440	Ulcer size, healing time, healing rate, cost effectiveness	AllogeneicIntralesional adipose-derived mesenchymal stem cells	DFU on any side of the foot (great toe, other toes, foot dorsum, forefoot, heel) with wound depth of Wagner grade 1 and 2 lesions
4	Thamm (2023) [21]	Germany	Randomized controlled trial	31	17	14	60	60	Wound size, pain level, bacterial colonisation and wound infection, histological assessment, neovascularization(CD31)	Sublesional fat grafting	Chronic ulcers due to diabtes and other causes
5	Smith (2020) [22]	UK	Randomized controlled trial	12	6	6	17	84	Wound size, wound healing, adverse events, cost implications, health-related quality of life, PUSH score	Fat grafting	DFU with ulcer area > 25 mm^2^ and < 10 000 mm^2^
6	Moon (2019) [20]	Korea	Randomized controlled trial	39	22	17	11	84	Ulcer size, healing time, healing rate	Allogeneic adipose-derived stem cell hydrogel complex	DFU of size between 1 and 25 cm^2^; and depth of Wagner grade 1 and 2

**Table 2:** Baseline characteristics of the included studies

No	Study ID	Age (years), mean (SD)		Gender, N. (%)				Heigh (cm), mean (SD)		Weigh (kg), mean (SD)		Body mass index, mean(SD)		Smoking, N. (%)		Duation of diabetes (Months), mean (SD)		Infected ulcer, N (%)	
				Male		Female													
		ADSCs	Control	ADSCs	Control	ADSCs	Control	ADSCs	Control	ADSCs	Control	ADSCs	Control	ADSCs	Control	ADSCs	Control	ADSCs	Control
1	Tanios (2021) [17]	48.12 (14.58)	48.04 (10.56)	29 (58%)	24 (48%)	21 (42%)	26 (52%)											10 (20%)	10 (20%)
2	Lonardi (2019) [18]	69 (11.6)	71.6 (10.8)	45 (79%)	41 (72%)	12 (21%)	16 (28%)							11 (20%)	8 (14%)				
3	Uzun (2021) [19]	57.5 (8.4)	57.2 (4.5)	6 (50%)	6 (50%)	4 (50%)	4 (50%)					27 (4)	26.7 (4)			160.8 (42)	174 (40.8)		
4	Thamm (2023) [21]	62 (11)	60 (14)	13 (76.5%)	13 (92.9%)	4 (23.5%)	1 (7.1%)	177 (9)	178 (5)	93 (21)	95 (22)	29.7 (6.68)	29.98 (6.94)	5 (29.4%)	7 (50%)			10 (61%)	13 (92%)
5	Smith (2020) [22]	60.2	55.2	6 (100%)	5 (83.3%)	0	1 (16.7%)					26.9	30.7		2 (33.3%)				
6	Moon (2019) [20]	59.9 (13.3)	68.4 (9.9)	14 (63.6%)	13 (76.5%)	8 (36.4%)	4 (23.5%)	167.9 (11.4)	167.3 (7.3)	72.9 (15.7)	73.3 (14.2)	25.8 (4.1)	26.2 (5)	5 (22.7%)	3 (17.6%)	205.7 (128)	239.7 (117.3)		

No	Study ID	Primary diseases, N. (%)								Duration of ulcer (weeks), Mean (SD)		Size of ulcer (cm2), Mean (SD)		Wagner grade of ulcer, N. (%)			
		Hypertenstion		cardiac disorder		Renal disorder		Neurological disorder						Grade 1		Grade 2	
		ADSCs	Control	ADSCs	Control	ADSCs	Control	ADSCs	Control	ADSCs	Control	ADSCs	Control	ADSCs	Control	ADSCs	Control
1	Tanios (2021) [17]									47.12 (37.52)	48.16 (29.96)	6.28 (0.98)	5.63 (0.75)				
2	Lonardi (2019) [18]	50 (88%)	50 (88%)	35 (61%)	43 (75%)	20 (35%)	28 (49%)	2 (4%)	8 (14%)								
3	Uzun (2021) [19]	4 (40%)	7 (70%)	5 (50%)	4 (40%)	2 (20%)		1 (10%)		7.89 (2.97)	6.84 (2.41)	23.5 (5.6)	25.8 (5.4)	6 (54.5%)	5 (45.5%)	4 (44.4%)	5 (55.6%)
4	Thamm (2023) [21]	9 (52.9%)	5 (35.7%)	2 (11.6%)	1 (7.1%)					105 (130)	106 (129)	6.3 (6.2)	4.8 (4.0)				
5	Smith (2020) [22]									41	49	3.1 (1.3)	6.4 (5.9)				
6	Moon (2019) [20]			18 (81.8%)	16 (94.1%)	7 (31.8%)	10 (58.8%)	13 (59.1%)	11 (64.7%)	28.7 (47.1)	43.7 (99.85)	2 (0.9)	2.8 (2)	14 (63.6%)	11 (64.7%)	8 (36.4%)	6 (35.3%)

### Risk of bias assessment

We used the Cochrane Risk of Bias tool (RoB2) for demonstrating the bias in our included studies. All studies exhibited a low risk of bias in the randomization process, deviations from intended interventions, and missing outcome data, indicating robust data integrity. For the measurement of the outcome, while most studies showed a low risk, Moon [20] demonstrated an unclear risk of bias. In the domain of selection of the reported result, all studies demonstrated a low risk of bias, ensuring comprehensive and transparent reporting. Overall, the quality of the included studies is considered high, with minor concerns regarding blinding in a few instances, particularly in the Moon [20] study. Risk of bias assessment and summary of the included studies domain is shown in Fig. 2.

The Grading of Recommendations Assessment, Development, and Evaluation (GRADE) system was used to investigate the level of the evidence. In this assessment, outcomes such as healing rate and overall healing state received a high certainty rating. This high certainty was supported by a consistent and substantial improvement in healing rates among patients treated with ADSCs across multiple studies. For example, at 6 weeks, ADSCs achieved significantly higher healing rates compared to standard care (RR 3.04, 95% CI 1.87–4.94).

Conversely, the certainty for the healing time outcome was rated as very low due to high heterogeneity and imprecision. The overall heterogeneity (I^2^ = 96%) indicated considerable variability among the studies, which was even more pronounced in the younger patient subgroup (I^2^ = 86%). Such variability and wide confidence intervals reduced the precision of the findings. Despite these limitations, the GRADE assessment highlights a high certainty in the favorable impact of ADSCs on healing rates and total healing state, although further research is warranted to address the imprecision in healing time outcomes, the details of the GRADE has been demonstrated in GRADE Evidence Table.

### Effect of ADSCs on diabetic foot ulcers healing

#### Total healing state

**Fig. 3:**
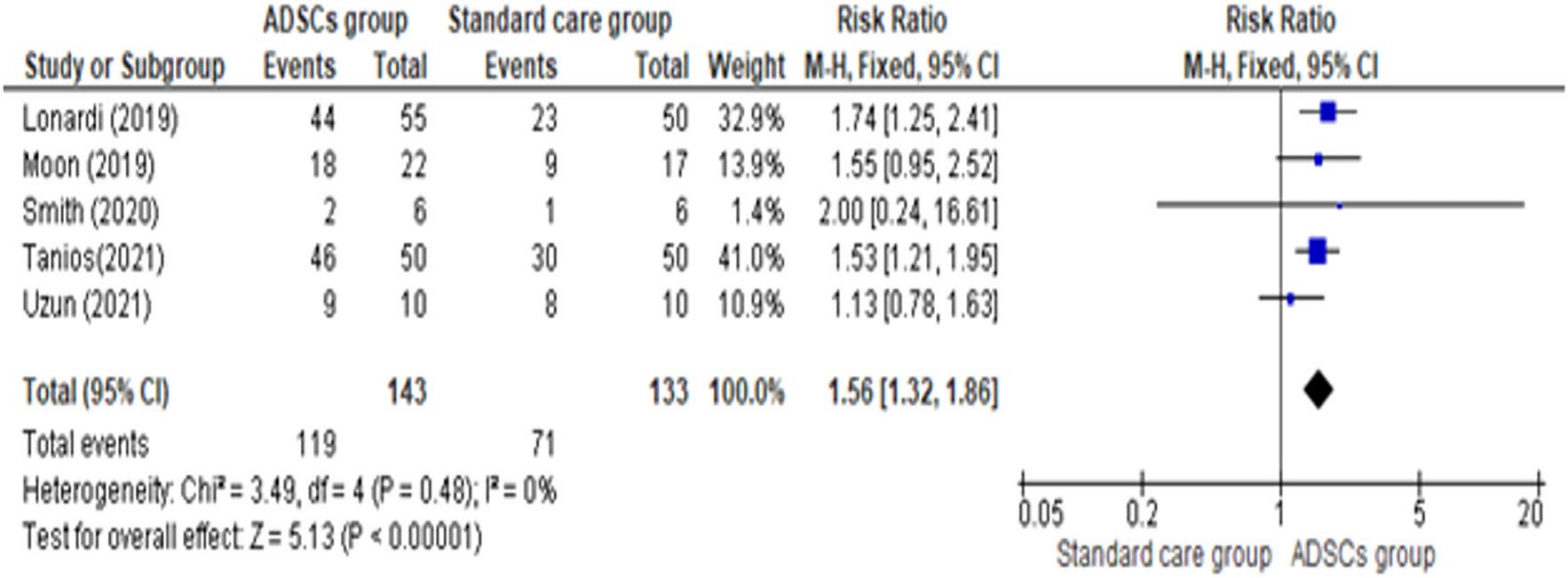
Comparison of the Total Healing State between the ADSCs group and the Standard care group at the end of follow-up period

As shown in Fig. 3, Five studies reported that more participants healed completely by the end of the follow-up durationin the ADSCs group compared with the Standard care group (Risk ratio = 1.56, 95% CI [1.32, 1.86], P < 0.00001). Pooled studies were homogenous (Chi-square P = 0.48; I^2^ = 0%), suggesting that Adipose Derived stem cell Intervention was related to improved total healing state.

#### Healing rate

**Fig. 4:**
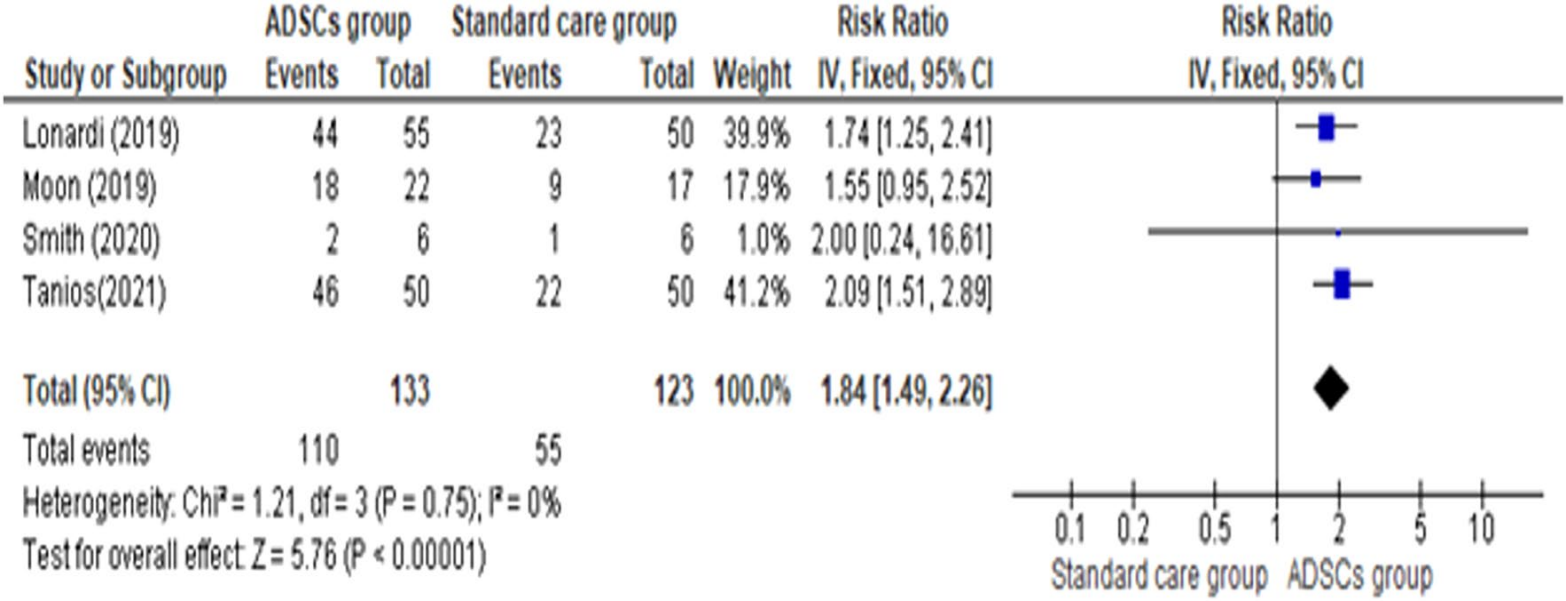
Comparison of the Healing Rate between the ADSCs group and the Standard care group at the end of follow-up

**Fig. 5:**
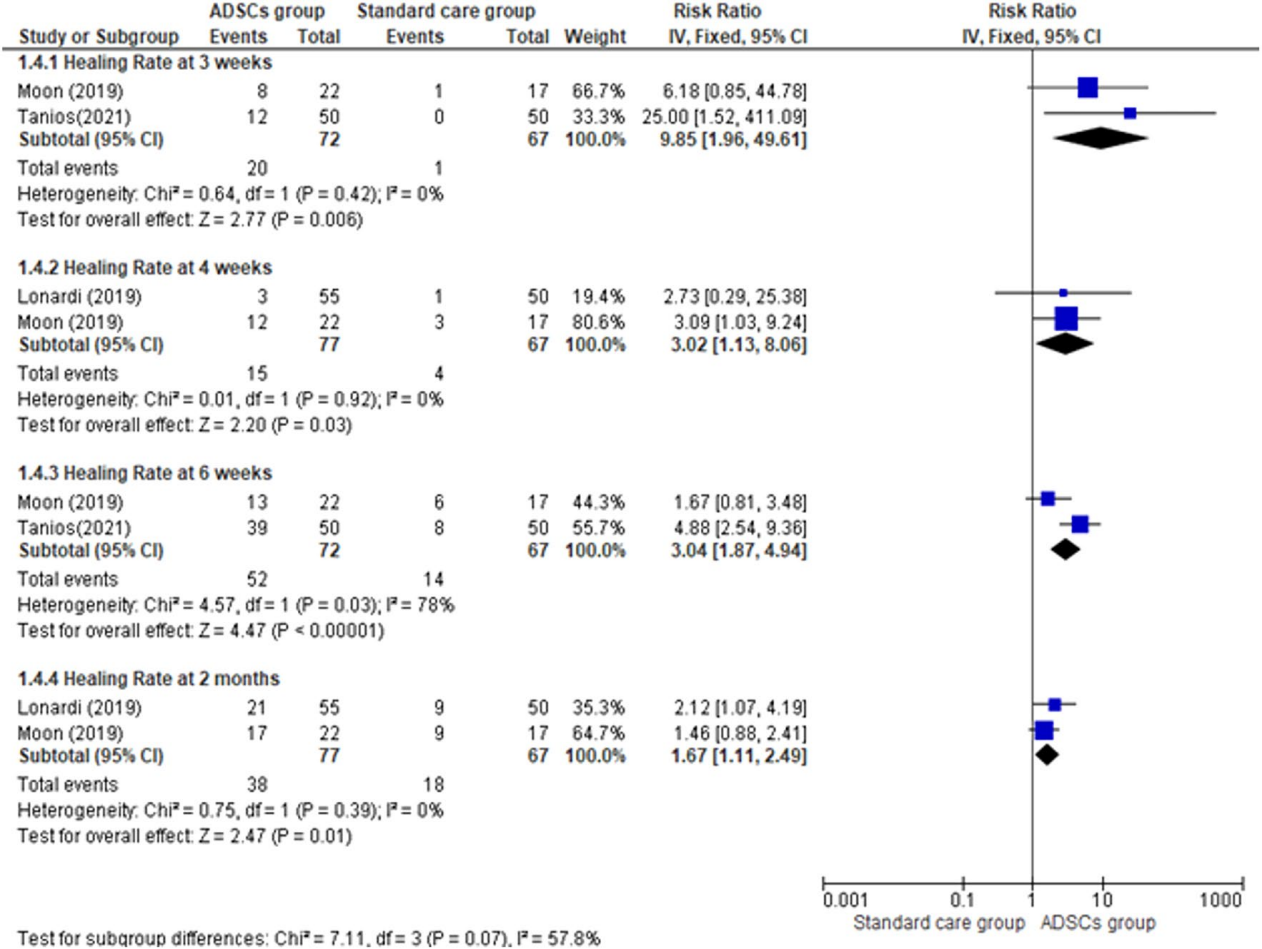
Comparison of the Healing Rate between the ADSCs group and the Standard care group at different follow-up points (3 weeks, 4 weeks, 6 weeks, 2 months)

As shown in Fig. 4, 5, The overall effect estimate favors the ADSCs group (pooled effect estimate = 1.84, 95% CI [1.51, 2.89], P < 0.00001). Results were consistent across the studies between the two groups (ADSCs group and Standard care group) (Chi-square P = 0.75; I^2^ = 0%). Subgroup analysis was done to investigate adipose-derived stem cells’ effect on the healing rate of diabetic foot ulcers at different follow-up points.

The ADSCs group healing rate was significantly higher than the Standard care group at the 3 weeks of follow-up (pooled effect estimate = 9.85, 95% CI [1.96, 49.61], P = 0.006), at the 4 weeks of follow-up (pooled effect estimate = 3.02, 95% CI [1.13, 8.06], P = 0.03), at 6-week period of follow-up (pooled effect estimate = 3.04, 95% CI [1.87, 4.94], P = 0.00001), and at 2-month period of follow-up (pooled effect estimate = 1.67, 95% CI [1.11, 2.49], P = 0.01). Pooled studies for all subgroups of follow-up periods were homogenous (Chi-square P > 0.1; I^2^ < 50%) except at 6 weeks of follow-up there was marked heterogeneity (Chi-square P = 0.03; I^2^ = 78%). This leads to inconsistent healing rate results among studies at different follow-up periods (test for subgroup differences: P = 0.07, I^2^ = 57.8%).

#### Healing time (days)

**Fig. 6:**
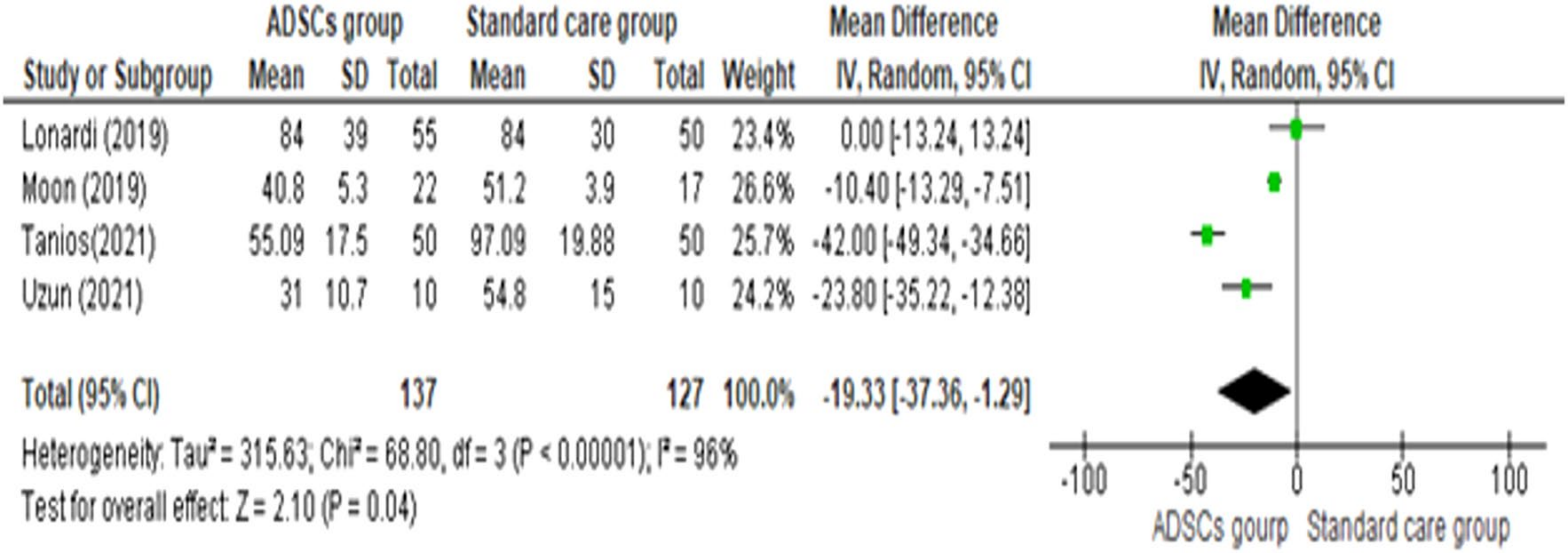
Comparison of the Total Healing Time (days) between the ADSCs group and the Standard care group

**Fig. 7:**
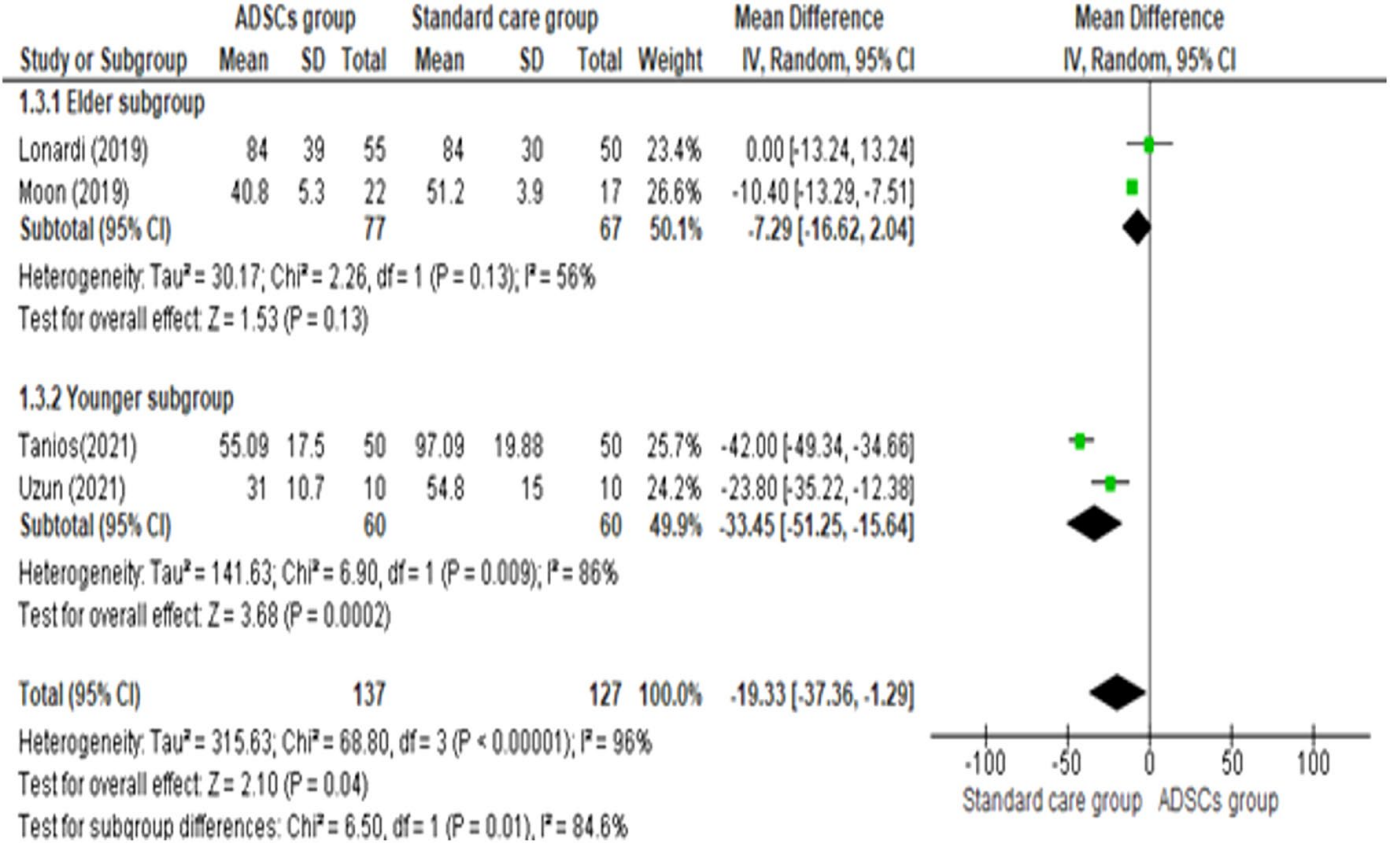
Comparison of the Total Healing Time (days) between age-based subgroups of the ADSCs group and the Standard care group

As shown in Fig. 6, 7, The pooled mean difference of the studies showed that the ADSCs group needed a smaller number of days to heal than the Standard care group (pooled mean difference = −19.33, 95% CI [−37.36, −1.29], P = 0.04). A random-effects model was employed to pool the data due to the high heterogeneity across the studies (Chi-square P < 0.00001; I^2^ = 96%).

To investigate the source of heterogeneity, subgroup analyses were performed. Heterogeneity was partially resolved with age-based subgroup analysis. The elder subgroup, which included Lonardi [18] and Moon [20] was homogenous (Chi-square P = 0.13; I^2^ = 56%) but the younger subgroup, which included Tanios [17] and Uzun [19] was heterogeneous (Chi-square P = 0.009; I^2^ = 86%). There is a significant difference in the healing time between the two subgroups. (Test for subgroup differences: P = 0.01, I^2^ = 84.6%).

## Discussion

This systematic review demonstrates the efficacy of ADSCs in diabetic foot ulcers versus standard wound care methods in terms of total Healing State (Risk ratio = 1.56, 95% CI [1.32, 1.86], P < 0.00001) the primary efficacy outcome in most of the studies reviewed, Five studies showed that participants in the Adipose adipose-derived stem Cells (ADSCs) group had a significantly higher complete healing rate compared to the Standard care group, The homogeneity of the pooled studies (Chi square P = 0.48; I2 = 0%) suggests consistent results across different studies, reinforcing the association between ADSC therapy and an improved total healing state. Regarding healing rate, the overall effect estimate strongly favors the ADSCs group, with a pooled effect estimate of 1.84 (95% CI [1.51, 2.89], P < 0.00001). This consistency across studies (Chi square P = 0.75; I2 = 0%) highlights the reliability of the findings. Subgroup analysis further emphasizes the efficacy of ADSCs at different follow-up points (3 weeks, 4 weeks, 6 weeks, 2 months).

Among the studies we selected, 4 studies applied autologous ADSCs on 130 patients totally [17, 18, 21, 22] and the other 2 studies used allogenic ADSCs with a total population of 32 patients [19, 20].

Regrading ulcer size, (Thamm et al. 2020) reported significant wound reduction in favor of ADSCs group at weeks 2 and weeks 3 (P = 0.014), (P = 0.0004) respectively) but the final measure at 2 months was insignificant (p = 0.081) [21]. (Moon et al. 2019) reported higher wound size reduction for ADSCs than control group at week 1 (P = 0.007), it was also significant at week 9 and week 12 [20]. Smith et al. [20] report change of ulcer size between week 0 to week 12, the mean of Ulcer size decreased from 6.4 to 3.4 cm^2^ in the Control group and from 3.1 to 1.4 cm^2^ in the ADSCs group [22].

As regards wound infection [19, 20, 22], excluded patients with active wound infection or any systemic infection from the study [19, 20, 22], moreover Lonardi et al. [18] considered wound infection a failure of wound healing [18]. On the other hand, Tanios et al. [17] reported that 20 patients had an infected ulcer at the beginning of the study, 10 on each group, while at the end of the study only three (6%) patients in the study group developed infection, whereas 14 (28%) patients in the control group developed infections [17]. Thamm stated that 96.2% of population have bacterial colonization verified by wound swabs [21].

Regarding histological assessment, Thamm et al. [21] reported that neovascularization was observed higher in the group receiving ADSCS, Tanios et al. [17] reported a better re-epithelization and formation of granulation tissue in ADSCs group [17, 21]. In another clinical trial involving 16 patients aimed at investigating the therapeutic effect of fat graft in comparison to routine care and fat graft application with platelet rich plasma on DFUs reported that there was better angiogenesis in the fat graft group [23].

Regarding the safety, ADSCs were safe in our included studies except for minor adverse events as 2 abdominal hematomas as reported in Tanios et al. [17], wound infection as reported in Thamm et al. [21] and also Pain level was reported in 2 studies [18, 21] as visual analogue scale (VAS) (1–10) and in another one [19] it was as a part of Short Form (SF-36) score but all of them stated no significant difference between two arms [18, 19, 21, 24, 25]. none of the 2 studies [19, 20] used allogeneic ADSCs reported any adverse effects related to immune reactions.

Quality of life was reported on different scales in 2 studies. In Uzun et al. [19], it was reported through SF-36-Physical functioning and SF-36-General health resulting in significant difference in favor of ADSCs group (P = 0.017, P = 0.010, respectively) [24]. Smith et al. [22] used a health-related quality of life (HRQOL) questionnaire resulting in a significantly lower score for the control group (P < 0.05). Also, in another cohort study done on Lonardi et al. [18] subject, it was concluded that ADSCs achieve a better quality of life and decrease the length of hospital stay [26, 27].

Regarding cost effectiveness [22], stated that there is no significant difference between two groups [22]. While [19] reported that ADMSCs group costs more than control group (6695.3 ± 329.0 USD, 4082.0 ± 979.8 USD, respectively (P = 0.001) but it may be cost effective modality due to its benefits in achieving early return to work and decreasing recurrence and amputation rate [19].

Regarding our study, 6 clinical trials were included, reported that more participants healed completely by the end of follow-up duration in the ADSCs group compared with the Standard care group, and for the healing time, ADSCs groups showed significantly lower healing time than the standard care groups. In spite of the methodological and clinical heterogeneity observed in our 6 included studies, our meta-analysis assumed that ADSCs could be a promising option for treatment of DFU as the 6 studies reported more healed patients at the end of follow up duration and lower healing time for ADSCs group.

Our study’s findings showed that ADSCs groups have a better healing rate and lower healing time, in addition to better overall healing results than standard care groups confirming that ADSCs have an advantage over standard care groups. However, Several limitations must be considered first standard care for DFUs varied widely across studies, complicating comparisons. Lonardi [18] used sodium hypochlorite and saline solution with paraffin gauze, emphasizing rest and immobilization [18]. Moon [20] used hydrogen peroxide and saline with polyurethane foam [20]. Smith [22] followed individualized clinical guidelines, while Tanios [17] employed local anesthesia, debridement, and betadine ointment every 3 weeks [17, 22]. Thamm [21] used saline injections with polyurethane dressings, and Uzun [19] employed conventional glucose regulation and negative pressure therapy [19, 21]. This variability in standard care methods presents a limitation, as the lack of standardization in cleaning, dressing, and offloading protocols hinders the establishment of unified treatment guidelines Furthermore, our findings are limited by the small number of reported outcomes and there is other data like ulcer size reduction, pain level, cost effectiveness and other outcomes that cannot be assessed within our statistical analysis due to insufficient data provided in the selected studies, Significant heterogeneity was noted in the finding reported. Therefore, we performed subgroup analyses to determine the cause of heterogeneity. Our studies also have high risk of bias in some domains due to the presence of some concerns regarding selective reporting and blinding which is unavoidable in most surgical intervention studies. Therefore, our results should be cited with caution.

## Recommendations

Our study recommends that further clinical trials targeting the usage of ADSCs in clinical settings are needed. Specifically, we recommend conducting more extensive clinical trials with larger groups of patients to better demonstrate the safety and effectiveness of ADSC therapy, it is important to demonstrate any possible adverse events or unfavorable outcomes that may be associated with ADSCs application.

## Conclusion

Our study concluded that ADSCs groups have multiple advantages over standard care groups. Patients in the ADSCs group showed higher rates of total healing, better healing rates, and shorter healing time compared to the control group. ADSCs appear to be safe but more clinical trials are needed to assess its adverse effects, additionally, ADSCs showed cost-effectiveness by decreasing hospital admission and achieving an early return to work compared to standard care groups.

The combined use of evidence from this systematic review underscores the critical importance of conducting thorough research to bridge the gap between theory and practice.

## Supplementary Information

Supplementary material 1.

## Data Availability

Data is provided within the manuscript or supplementary information files.

## Abbreviations

IDF: International Diabetes Federation
ADSCs: ADSCs adipose-derived stem cells
RoB: Cochrane risk of bias tool
RevMan: Review manager
MD: Mean difference
CI: Confidence interval
DM: Diabetes mellitus
DFUs: Diabetic foot ulcers
MSCs: Mesenchymal stem cells
VEGF: Vascular endothelial growth factor
TNF- α: Tumor Necrosis Factor-alpha
PRISMA: Preferred Reporting Items for Systematic Reviews and Meta-analyses
