# Supplementary material for: Therapeutic potential of adipose-derived stem cells for diabetic foot ulcers: a systematic review and meta-analysis
Source: Diabetol Metab Syndr. 2025 Jan 7;17:9. doi: 10.1186/s13098-024-01523-5 (PMC11706097; doi:10.1186/s13098-024-01523-5)
Supplement: Supplementary file 1 — Supplementary material 1. [file 13098_2024_1523_MOESM1_ESM.docx]

**Author(s):**

**Question:** ADSCs group compared to Standard Care group for [Patients with diabetic foot ulcers]

**Setting:**

**Bibliography:** . [Adipose stem cells] for [Diabetic foot ulcers]. Cochrane Database of Systematic Reviews [Year], Issue [Issue].

| **Certainty assessment** | | | | | | | **№ of patients** | | **Effect** | | **Certainty** | **Importance** |
| --- | --- | --- | --- | --- | --- | --- | --- | --- | --- | --- | --- | --- |
| **№ of studies** | **Study design** | **Risk of bias** | **Inconsistency** | **Indirectness** | **Imprecision** | **Other considerations** | **ADSCs group** | **Standard Care group** | **Relative (95% CI)** | **Absolute (95% CI)** |  |  |
| **Total Healing State** | | | | | | | | | | | | |
| 5 | randomised trials | not serious | not serious | not serious | not serious | none | 119/143 (83.2%) | 71/133 (53.4%) | **RR 1.56** (1.32 to 1.86) | **299 more per 1,000** (from 171 more to 459 more) | ⨁⨁⨁⨁ High |  |
| **Healing time (Days)** | | | | | | | | | | | | |
| 4 | randomised trials | not serious | very serious^a^ | not serious | serious^b^ | none | 137 | 127 | - | MD **19.33 lower** (37.36 lower to 1.29 lower) | ⨁◯◯◯ Very low^a,b^ |  |
| **Healing time (Days) - Elder subgroup** | | | | | | | | | | | | |
| 2 | randomised trials | not serious | very serious^a^ | not serious | serious^b^ | none | 77 | 67 | - | MD **7.29 lower** (16.62 lower to 2.04 higher) | ⨁◯◯◯ Very low^a,b^ |  |
| **Healing time (Days) - Younger subgroup** | | | | | | | | | | | | |
| 2 | randomised trials | not serious | very serious^a^ | not serious | serious^b^ | none | 60 | 60 | - | MD **33.45 lower** (51.25 lower to 15.64 lower) | ⨁◯◯◯ Very low^a,b^ |  |
| **Healing Rate** | | | | | | | | | | | | |
| 4 | randomised trials | not serious | not serious | not serious | not serious | none | 110/133 (82.7%) | 55/123 (44.7%) | **RR 1.84** (1.49 to 2.26) | **376 more per 1,000** (from 219 more to 563 more) | ⨁⨁⨁⨁ High |  |
| **Healing Rate - Healing Rate at 3 weeks** | | | | | | | | | | | | |
| 2 | randomised trials | not serious | not serious | not serious | not serious | none | 20/72 (27.8%) | 1/67 (1.5%) | **RR 9.85** (1.96 to 49.61) | **132 more per 1,000** (from 14 more to 726 more) | ⨁⨁⨁⨁ High |  |
| **Healing Rate - Healing Rate at 4 weeks** | | | | | | | | | | | | |
| 2 | randomised trials | not serious | not serious | not serious | not serious | none | 15/77 (19.5%) | 4/67 (6.0%) | **RR 3.02** (1.13 to 8.06) | **121 more per 1,000** (from 8 more to 421 more) | ⨁⨁⨁⨁ High |  |
| **Healing Rate - Healing Rate at 6 weeks** | | | | | | | | | | | | |
| 2 | randomised trials | not serious | not serious | not serious | not serious | none | 52/72 (72.2%) | 14/67 (20.9%) | **RR 3.04** (1.87 to 4.94) | **426 more per 1,000** (from 182 more to 823 more) | ⨁⨁⨁⨁ High |  |
| **Healing Rate - Healing Rate at 2 months** | | | | | | | | | | | | |
| 2 | randomised trials | not serious | not serious | not serious | not serious | none | 38/77 (49.4%) | 18/67 (26.9%) | **RR 1.67** (1.11 to 2.49) | **180 more per 1,000** (from 30 more to 400 more) | ⨁⨁⨁⨁ High |  |

**CI:** confidence interval; **MD:** mean difference; **RR:** risk ratio

#### Explanations

a. The overall heterogeneity is extremely high (I² = 96%, χ² P < 0.00001). Subgroup analysis indicates moderate heterogeneity in the elder group (I² = 56%, χ² P = 0.13) and high heterogeneity in the younger group (I² = 86%, χ² P = 0.009). The significant difference between subgroups (P = 0.01, I² = 84.6%) suggests age-related factors may contribute to the variability in healing time across studies.

b. The healing time outcome shows a wide confidence interval (-37.36 to -1.29 days), indicating a statistically significant reduction but with uncertainty about the exact effect size. High heterogeneity (I² = 96%) suggests considerable variability between studies, further impacting precision. Subgroup analyses showed moderate heterogeneity in older participants (I² = 56%) and high heterogeneity in younger participants (I² = 86%). These factors together indicate a moderate risk of imprecision for healing time.
